# Patterns of gestational diabetes diagnosis inside and outside of clinical guidelines

**DOI:** 10.1186/s12884-016-1191-6

**Published:** 2017-01-06

**Authors:** Jacinda M. Nicklas, Chloe A. Zera, Janet Lui, Ellen W. Seely

**Affiliations:** 1 Division of General Internal Medicine, University of Colorado School of Medicine, 12348 E. Montview Blvd, C263, Aurora, CO 80045 USA; 2 Division of Maternal-Fetal Medicine, Department of Obstetrics and Gynecology, Brigham and Women’s Hospital, Harvard Medical School, 75 Francis Street, Boston, MA 02115 USA; 3 Division of Endocrinology, Diabetes and Hypertension, Brigham and Women’s Hospital, Harvard Medical School, 221 Longwood Avenue, Boston, MA 02115 USA

**Keywords:** Gestational diabetes, Pregnancy, Diagnosis, Guidelines, Discharge codes

## Abstract

**Background:**

Hospital discharge codes are often used to determine the incidence of gestational diabetes mellitus (GDM) at state and national levels. Previous studies demonstrate substantial variability in the accuracy of GDM reporting, and rarely report how the GDM was diagnosed. Our aim was to identify deliveries coded as gestational diabetes, and then to determine how the diagnosis was assigned and whether the diagnosis followed established guidelines.

**Methods:**

We identified which deliveries were coded at discharge as complicated by GDM at the Brigham and Women’s Hospital in Boston, MA for the year 2010. We reviewed medical records to determine whether the codes were appropriately assigned.

**Results:**

Of 7883 deliveries, coding for GDM was assigned with 98% accuracy. We identified 362 cases assigned GDM delivery codes, of which 210 (58%) had oral glucose tolerance test (OGTT) results available meeting established criteria. We determined that 126 cases (34%) received a GDM delivery code due to a clinician diagnosis documented in the medical record, without an OGTT result meeting established guidelines for GDM diagnosis. We identified only 15 cases (4%) that were coding errors.

**Conclusions:**

Thirty four percent of women assigned GDM delivery codes at discharge had a medical record diagnosis of GDM but did not meet OGTT criteria for GDM by established guidelines. Although many of these patients may have met guidelines if guideline-based testing had been conducted, our findings suggest that clinician diagnosis outside of published guidelines may be common. There are many ramifications of this approach to diagnosis, including affecting population-level statistics of GDM prevalence and the potential impact on some women who may be diagnosed with GDM erroneously.

## Background

Hospital discharge data are commonly used to estimate the prevalence of pregnancy complications including gestational diabetes mellitus (GDM). These prevalence data have the potential for far-reaching impact, since they are often used at local and national levels to influence maternal and child health programs, monitor trends, and determine allocation of resources [1]. Therefore, understanding the patterns by which GDM diagnoses are given is important. Previous studies demonstrate variable accuracy for the diagnosis of GDM during pregnancy as reported by hospital discharge data [2–4]. Of note, many studies use documentation of GDM in the medical record as the “gold standard,” but few studies have examined how the GDM diagnosis was made [5, 6].

In 2010, the obstetric services at the Brigham and Women’s Hospital (BWH) were using the American College of Obstetricians and Gynecologists (ACOG) criteria to diagnose GDM. These criteria were first established in 2001 and then reaffirmed in 2013 [7, 8]. These guidelines recommend that all pregnant women be screened for diabetes with a 50-g glucose load test (GLT) between 24 and 28 weeks of gestation. The recommendations state that women with a GLT result of 130 or 140 mg/dL (7.2 or 7.8 mmol/L) or greater should undergo a three hour 100-g oral glucose tolerance test (OGTT), with a diagnosis of GDM given for two or more abnormal values based on Carpenter-Coustan criteria [7, 9]. The degree to which GDM diagnosis is made outside of these published guidelines has not been quantified nor well-described, yet may have significant impact on the integrity of reported data. We therefore sought to validate the hospital discharge diagnosis of GDM by comparing it to the medical record diagnosis of GDM. We then compared the medical record diagnosis to ACOG criteria to determine whether the diagnosis of GDM was made according to established guidelines.

## Methods

The BWH is the largest obstetrical service in New England with over 6000 deliveries per year, and approximately 5% of these pregnancies are complicated by GDM. In 2010, at discharge, a delivery complicated by GDM was assigned codes 648.80 (Diabetes mellitus of mother complicating pregnancy childbirth or the puerperium unspecified as to episode of care), 648.81 (Diabetes mellitus of mother with delivery) and/or 648.83 (Antepartum diabetes mellitus), according to the International Classification of Diseases, Ninth Revision (ICD-9). We used the Research Patient Data Registry (RPDR), a centralized database that houses clinical data from patient medical records within the Partners Healthcare system, to obtain lists of patients discharged between January 1, 2010 and December 31, 2010 with at least one of the ICD-9 discharge codes associated with a diagnosis of GDM. In addition, we obtained a case list of all women discharged with at least one of the ICD-9 codes associated with a diagnosis of type 1 diabetes or type 2 diabetes (ICD-9 codes 648 and 250) so that we could check for any deliveries to women with GDM that may have been miscoded. We then used the RPDR to obtain a separate list for lab values of all 3-h OGTTs performed at BWH between March 1, 2009 and December 31, 2010. Since the 3-h OGTT test is used only during pregnancy to diagnose GDM, identifying these tests enabled us to create an additional case list to compare with the other lists. The institutional review board at BWH approved this project and a waiver of consent was received from the Partners Human Research Committee to conduct the medical record review.

Once we identified cases of potential GDM either by ICD-9 delivery code or by laboratory results, we reviewed medical records to obtain age, race, parity, history of prior GDM, use of insulin during pregnancy, mode of delivery, gestational age at delivery, pre-pregnancy body mass index (BMI), and infant birth weight, as well as results of GLT and OGTT testing. For cases where laboratory results for the GLT or OGTT were not available within the BWH system, we reviewed results in clinic notes and outside laboratory reports when available.

Once we obtained all of the ICD-9 coded cases as well as all available GLT and OGTT values, we compared the laboratory values to the Carpenter-Coustan ACOG guidelines [8, 9]. We then placed cases into one of five categories: 1) diagnosed by guidelines, 2) diagnosed outside of guidelines, 3) clinician error, 4) coding error, or 5) no laboratory values available (Table 1). A case met the criteria for diagnosis by guidelines if the 3-h OGTT had at least two abnormal values based on the Carpenter-Coustan thresholds and the GDM ICD-9 code was properly assigned. If the pre-pregnancy or first trimester HbA1c met criteria for type 2 diabetes, we considered these cases to have preexisting diabetes and therefore defined as a clinician error. For cases not meeting the Carpenter-Coustan laboratory criteria, we reviewed the medical record to determine whether and how a clinician made a diagnosis of GDM. A case qualified as “GDM diagnosed outside of guidelines” if the laboratory results in the medical record did not meet criteria, but the clinician provided treatment for GDM, including a prescribed diet, oral medication, and/or insulin. Two physicians independently reviewed the cases not meeting diagnosis criteria by guidelines, as well as the records of cases assigned ICD-9 codes for T1DM (*n* = 13) or T2DM (*n* = 29) to ensure that none of these cases should have been coded as GDM deliveries.

**Table 1:** Categories for ICD-9 GDM Discharge Diagnoses

Category	Criteria
Diagnosed by guidelines	3-h OGTT with ≥2 abnormal values based on Carpenter-Coustan criteria
Diagnosed outside of guidelines	Clinician treated pregnancy as GDM due to one or more of the following:
	-GLT ≥ 200 mg/dL (11.1 mmol/L)
	-History of GDM or IFG or IGT
	-1 abnormal value on OGTT
	-Elevated fingersticks
	-Inability to tolerate GLT or OGTT
	-Elevated lab value (HbA1c, fasting or random blood glucose)
Clinician error	-Clinician misread OGTT results
	-Type 2 DM called GDM
Coding error	ICD-9 code assigned and no mention of GDM diagnosis or treatment in prenatal care notes, delivery admission, anesthesia record, or discharge summary
	OR 3 h OGTT ≥2 abnormal values with GDM diagnosis in chart and no ICD-9 code assigned
No laboratory values available	Transferred to BWH with GDM diagnosis and no laboratory values or provider mentioned test done but no values available

We compiled descriptive tables of cases in the five different categories, and then compared those GDM cases diagnosed by guidelines with those diagnosed outside of guidelines using univariate and multivariate analyses to determine predictors of diagnosis outside of guidelines, using a significance level of *p* < .05. Univariate analyses included t-tests, chi-squared tests, Fisher’s Exact tests, and Wilcoxon tests, as appropriate. Multivariable logistic regression analyses using variables with *p* values less than .20 were also performed. Analyses were conducted using JMP 10 Pro.

## Results

**Fig. 1:**
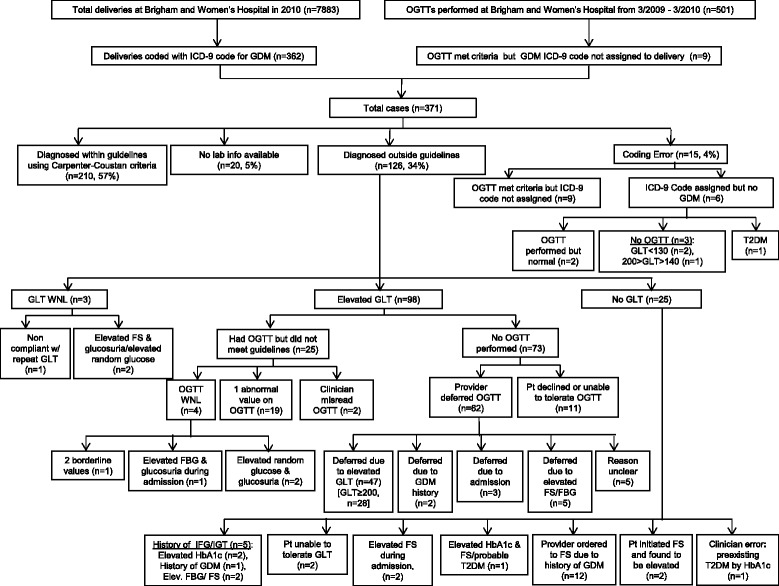
Patterns of gestational diabetes diagnoses for deliveries at Brigham and Women’s Hospital in 2010. OGTT = Oral Glucose Tolerance Test; GDM = Gestational Diabetes Mellitus; ICD-9 = International Classification of Diseases, Ninth Revision; GLT = Glucose Load Test (50-g); T2DM = Type 2 Diabetes Mellitus; WNL = Within Normal Limits; FS = fingersticks; FBG = fasting blood glucose; IFG = Impaired Fasting Glucose; IGT = Impaired Glucose Tolerance; HbA1c = glycated hemoglobin

Out of 7883 total deliveries, 362 (4.6%) cases of GDM were identified by ICD-9 discharge coding (Fig. 1). We validated 210 cases (57% of all GDM cases) as appropriately coded and as diagnosed within guidelines based on laboratory values meeting Carpenter-Coustan criteria. An additional 126 GDM cases (34%) were diagnosed outside of guidelines. Three of these cases were determined to be clinician error. In these cases, the GDM code was assigned appropriately by the coders since the diagnosis was clearly specified in the medical record; however the GDM was incorrectly diagnosed by the clinician. In addition, we identified 9 cases (0.1% of all deliveries) where the OGTT met criteria but the ICD-9 delivery code for GDM was not assigned. We identified 6 cases (0.08% of all deliveries) where a GDM diagnosis was assigned but either there were no laboratory data meeting criteria, or the patient had documented Type 2 diabetes. There were 20 cases (5.5% of GDM discharge codes) where laboratory data were unavailable from the medical record and we could not assess the validity of the assigned delivery code. All deliveries coded as type 1 diabetes and type 2 diabetes were appropriately assigned.

As noted, clinical diagnosis of GDM outside of published guidelines was common (*N* = 126, 34% of GDM cases). There were multiple reasons for clinicians to diagnose women with GDM outside guidelines (Fig. 1). Of the 126 cases diagnosed outside of guidelines, 101 had some form of glucose tolerance testing (80%). An elevated GLT value alone led to diagnosis of GDM in 47 cases (37%), of which 28 (22%) of these cases had a GLT result ≥ 200 mg/dL (11.1 mmol/L). Nineteen of the 126 (15%) were treated as GDM due to an elevated GLT and only one abnormal value on the OGTT. In 25 cases no glucose tolerance testing was performed. Twelve of these women had a history of GDM and the clinician initiated treatment without any formal glucose testing.

Characteristics of the cases diagnosed by guidelines and the cases diagnosed outside guidelines are shown in Table 2. Age, race, and pregnancy outcomes did not differ significantly between the two groups. Women diagnosed by guidelines were more likely to be nulliparous (*N* = 108, 51%) than those diagnosed outside of guidelines (*N* = 40, 32%, *P* = .0004). Similarly, women diagnosed by guidelines were less likely to have a history of GDM than women diagnosed outside of guidelines (*N* = 31 (15%) vs. 57 (45%), *P* < .0001). Among the 88 women with a history of GDM, only 31 (35%) were diagnosed by guidelines (as compared to 57% overall). In a logistic regression analysis, a history of GDM led to 4.7 (95% CI 2.8–7.9) increased odds of a diagnosis outside of guidelines. Insulin use was not retained in the multivariable model and nulliparity was removed from the model due to collinearity with “history of GDM.”

**Table 2:** Comparison of cases diagnosed by guidelines versus cases diagnosed outside of guidelines

Characteristic	Diagnosed by guidelines (*n* = 210)	Diagnosed outside of guidelines (*n* = 126)	*P* value for univariate analysis
Age in years, mean (SD)	34 (5)	34 (5)	0.69
Nulliparous, N (% of category)	108 (51%)	40 (32%)	0.0004
Race, N (% of category)			
Hispanic	42 (20%)	19 (15%)	0.30
Black	42 (20%)	26 (21%)	0.89
Asian	32 (15%)	26 (21%)	0.23
White	79 (38%)	51 (40%)	0.72
Other/not reported	15 (7%)	4 (3%)	
Prior GDM, N (% of category)	31 (15%)	57 (45%)	<.0001
Pre-pregnancy BMI kg/m^2^, median (25th%ile; 75th%ile) (*n* = 313)	27.5 (24, 33)	26.6 (24, 34)	0.82
BMI at delivery kg/m^2^, median (25th%ile; 75th%ile) (*n* = 336)	32.6 (28, 37)	31.8 (29, 38)	0.70
Pregnancy outcomes:			
Required insulin, N (% of category)	111 (53%)	78 (62%)	0.11
C-section, N (% of category)	92 (44%)	59 (47%)	0.59
Gestational age at delivery in weeks median (25th%ile; 75th%ile)	39 (37, 39)	38 (37, 39)	0.26
Infant birth weight gm, mean (SD)	3210 (661)	3209 (789)	0.96

## Discussion

In this study, we validated cases of deliveries assigned ICD-9 GDM discharge codes and found that 356/362 (98%) of cases assigned a GDM ICD-9 discharge code had GDM specified in the medical record. We identified only nine cases of deliveries with laboratory tests meeting criteria for GDM that should have been assigned a GDM ICD-9 code that were not. Of interest, we noted a large proportion of the GDM cases (34%) were diagnosed outside of established clinical guidelines. A history of GDM in a previous pregnancy demonstrated increased odds for a diagnosis outside of guidelines.

The high rate of accuracy (98%) seen in our study for GDM delivery codes may be in part due to the fact that GDM codes were assigned based on a thorough review of the medical record by experienced coders. In comparison, a medical record review study of deliveries in Washington State reported a true positive rate for GDM delivery discharge codes of 81% when compared to a review of the medical record [2]. In a medical record review study conducted among high volume hospitals in California, the sensitivity was 78% for GDM ICD-9 codes when compared to a review of the medical record [4].

In our study, we explored the way in which women were given a diagnosis of GDM and notably one-third of the GDM cases were diagnosed outside of established clinical guidelines. Of these, more than half had some abnormality in their GLT and/or OGTT, including 22% with a GLT greater than or equal to 200 mg/dl. Many of these women may have met criteria if guideline-based diagnosis had been conducted. However, a previous study revealed that, even with a cut-off of greater than or equal to 200 mg/dl on the 50 g GLT, the likelihood of GDM on a 3-h OGTT was only 69% [10]. Assigning a diagnosis of GDM to women with lesser degrees of glucose intolerance may reflect the clinician’s belief that these women would benefit from treatment. In the observational Hyperglycemia and Adverse Pregnancy Outcomes study, women with hyperglycemia not meeting criteria for GDM were still at increased odds of multiple adverse outcomes, including large-for-gestational-age (LGA) birthweight, high cord-blood serum C-peptide levels, primary cesarean delivery, neonatal hypoglycemia, premature delivery, shoulder dystocia or birth injury, intensive neonatal care, hyperbilirubinemia, and preeclampsia [11]. Despite the compelling observational data however, there is still no clear evidence that treatment of women with these milder glucose abnormalities will decrease rates of complications [12]. For this reason, at the time of the study and to date, the BWH has elected to use two-step testing for the diagnosis of GDM following Carpenter-Coustan criteria.

Diagnosis of GDM outside of guidelines may lead to falsely inflated estimates of recurrence risk, and also increase variability of administrative datasets. Consequently, the overall reliability of these datasets is decreased and may affect public health efforts and fund allocation. In addition, a diagnosis of GDM has many ramifications for women, including the need for frequent fingersticks, dietary restriction, a more medicalized pregnancy, and possibly increased psychological distress [13, 14]. Some studies suggest that just carrying a diagnosis of GDM increases the rate of cesarean delivery [8, 15, 16]. In addition, over-diagnosis of GDM is associated with system-wide costs. A pregnancy affected by GDM has an 18–34% increased cost over a normal pregnancy, at an average of $3305, an annual cost of $636 million [17].

An important finding in our study is that a history of GDM in a prior pregnancy was significantly associated with clinicians making a diagnosis of GDM without an OGTT meeting criteria by guidelines or sometimes without performing glucose tolerance testing at all. The tendency not to formally retest women with prior GDM may have a major impact on the estimates of recurrence of GDM as many studies of recurrence rates use a discharge diagnosis of GDM as the documentation for recurrence. Estimates of the rates of recurrence of GDM in a subsequent pregnancy are estimated at 41% from large population studies, with a broader range of 30-84% seen in studies of smaller populations [18, 19]. A recent report of recurrent GDM in Massachusetts looked at recurrent GDM using both discharge diagnosis and birth certificate data found similarly high rates of recurrence whether by birth certificate alone (38%), discharge diagnosis alone (47%) or the combination (48%). Importantly, however, the authors did not evaluate OGTT data [20]. Our study provides important insight into the data on the recurrence of GDM by documenting that at our institution only 35% of women with a history of GDM given a discharge diagnosis of GDM were diagnosed by an OGTT meeting criteria in established guidelines.

Our findings must be interpreted in the context of study design. We may not have identified all cases that should have been coded as GDM but were not, especially if no laboratory data were found in our system. This was a single institution study, and the coding and clinician diagnosis patterns we identified may not be representative of patterns at other hospitals.

Future studies should address the prevalence of GDM diagnosis outside of established guidelines in other settings, including community hospitals in addition to academic centers. Given recent controversy about new diagnostic guidelines for GDM [21], it is important to note that a large proportion of diagnoses may be made outside of guidelines. Estimates of the impact of the new guidelines on increasing the diagnosis of GDM [21] may substantially underestimate the true impact of more liberal guidelines on the incidence of GDM if clinical diagnosis outside of guidelines continues to occur. It may be important to clarify clinical coding rules and diagnosis guidelines in conjunction with the implementation of new criteria, particularly if clinicians are using their own judgment about when to apply guideline criteria. In addition, public health agencies that use ICD discharge data to estimate state and national incidence should be aware of the potential contribution of clinician diagnosis to those women receiving a chart diagnosis of GDM and therefore an ICD code of GDM.

## Conclusions

In this study, 98% of deliveries assigned ICD-9 gestational diabetes (GDM) discharge codes did have a diagnosis of GDM specified in the medical record. However, 34% of women assigned GDM delivery codes at discharge did not meet OGTT criteria for GDM by established guidelines. Although many of these patients may have met guidelines if guideline-based testing had been conducted, our findings suggest that clinician diagnosis outside of published guidelines may be common. There are many ramifications of this approach to diagnosis, including affecting population-level statistics of GDM prevalence and recurrence, as well as the potential impact on some women who may be diagnosed with GDM erroneously.

## Abbreviations

GDM: Gestational Diabetes Mellitus
GLT: Glucose Load Test (50-g)
HbA1c: Glycated hemoglobin
ICD-9: International Classification of Diseases, Ninth Revision
OGTT: Oral Glucose Tolerance Test
